# Quality of Chronic Care Statistically Mediates the Association Between eHealth Literacy and Self-Management Among Adults with Diabetes in a Shared Care Model

**DOI:** 10.3390/healthcare14172792

**Published:** 2026-09-01

**Authors:** Jinmei Su, Jing Cui, Xia Wang, Zhitian Cao, Mengchi Li, Ying Pang, Wei Cui, Wenhui Jiang, Ting Huang

**Affiliations:** 1 Department of Nursing, The First Affiliated Hospital, Xi’an Jiaotong University, Xi’an 710061, China; jinmeisu@stu.xjtu.edu.cn; 2 School of Nursing, Health Science Center, Xi’an Jiaotong University, Xi’an 710061, China; panda19950905@stu.xjtu.edu.cn (J.C.); mengchili@xjtu.edu.cn (M.L.); 3 Department of Geriatric Endocrinology and Metabolism, International Medical Ward 1, The First Affiliated Hospital, Xi’an Jiaotong University, Xi’an 710061, China; 000527@stu.xjtu.edu.cn (X.W.); 315142@stu.xjtu.edu.cn (Z.C.); 954524740@xjtu.edu.cn (Y.P.); 19503882057@stu.xjtu.edu.cn (W.C.)

**Keywords:** self-management, diabetes, eHealth literacy, quality of chronic care

## Abstract

Background/Objectives: As a lifelong chronic condition, diabetes requires self-management, which plays an essential role in slowing disease progression and improving treatment outcomes. This research aimed to investigate whether the quality of chronic care statistically mediates the relationship between eHealth literacy and self-management among adults with diabetes participating in a shared care model. Methods: A cross-sectional design along with convenience sampling was utilized in this study. From March 2025 to March 2026, 332 individuals with diabetes were enrolled from a shared care model at a tertiary hospital in northwestern China. The participants completed the Chinese versions of the three validated instruments, and the data were processed using SPSS 27.0 and PROCESS 4.1. Results: Significant positive correlations were found among the three core variables (all *p* < 0.01). The mediation model confirmed that the quality of chronic care acted as a partial and statistically significant mediating role in the relationship between eHealth literacy and self-management among adults with diabetes (β = 0.146, 95% CI: 0.114–0.398). Conclusions: This study found that patients with diabetes participating in the shared care model reported relatively favorable self-management capabilities. eHealth literacy was indirectly associated with self-management behaviors through the quality of chronic care. However, given the cross-sectional design of this study, these findings should be interpreted as statistical associations rather than causal evidence, and reverse causality cannot be ruled out. Future longitudinal studies are needed to establish temporal ordering and causal relationships among these variables.

## 1. Introduction

With accelerated population aging, lifestyle changes, and a rising proportion of overweight and obese individuals, the global prevalence of diabetes continues to rise. Consequently, diabetes has become a major public health concern worldwide, posing substantial challenges to health systems in both developed and developing countries [1,2]. According to data from the 11th (2025) IDF Diabetes Atlas, roughly 589 million adults worldwide had diabetes in 2025, with China accounting for around 148 million—the largest number globally [3]. Given its high incidence, low control rate, and low public awareness, diabetes not only undermines patients’ well-being and elevates the likelihood of mortality and disability but also imposes considerable economic burdens on health systems globally [4]. In 2024, the worldwide diabetes-related healthcare expenditure exceeded 1.015 trillion for the first time, representing 11.9% of total global healthcare spending [5]. Meanwhile, China’s direct expenditures on preventing and treating diabetes and managing complications are projected to rise from approximately $250 billion in 2020 to around $460 billion by 2030 [6].

Diabetes self-management refers to a set of proactive behaviors undertaken by patients to control disease progression, including monitoring blood glucose, adhering to medications, managing diet, engaging in regular physical activity, and practicing foot care [7]. These behaviors aim to monitor changes in the condition and to maintain or improve overall health. A systematic review has stated that advancing self-management capacity in individuals with long-term illnesses is a highly effective intervention in disease prevention and control [8]. Proficient self-management is critical for patients with diabetes to achieve glycemic control, enhance prognosis, and avoid both acute and chronic complications [9,10]. However, diabetes self-management among individuals in China is generally not ideal, an is characterized by low levels of awareness, treatment, and glycemic control being widespread [11]. Nearly half of all patients fail to achieve target blood glucose levels, making them highly susceptible to various acute and chronic complications [12].

eHealth literacy encompasses an individual’s skills in effectively seeking, accurately interpreting, critically evaluating, and appropriately utilizing digital resources to address health-related problems [13]. With the widespread adoption of digital healthcare and internet-based health services [14], eHealth literacy has effectively narrowed the communication gap between patients and healthcare providers [15], significantly enhancing the accessibility and convenience of health management, and demonstrating considerable potential for improving diabetes care quality and patients’ self-management capabilities [16]. This implies that individuals need digital skills to acquire, process, understand, and apply disease-related information [17]. eHealth literacy is the core expression of this capacity, and it has become an essential skill enabling patients to access high-quality care services and effectively manage their health themselves. Existing research indicates that greater eHealth literacy corresponds to improved health-related behaviors, self-management capabilities, and medication adherence among patients [18,19,20].

The quality of chronic care refers to patients’ subjective perception of how well the healthcare services they receive align with the chronic care model (CCM) framework [21]. The CCM provides a patient-centered approach to promote high-quality chronic disease care and collaborative patient–provider management [22]. To incorporate the latest developments in digital health, the eHealth-enhanced version of the CCM utilizes telemedicine and mobile health tools to support patients’ self-management [23]. To evaluate the practical effectiveness of the CCM, prior research has recommended measuring the quality of chronic care from the patient’s perspective [21], which aligns with patient experience indicators proposed by a leading international economic organization [24]. Existing research has demonstrated that the chronic care model can improve patients’ adherence to medication and glycemic control, enhance their self-management abilities, and reduce hospital readmission rates and medical expenditures, thereby leading to better long-term health outcomes [25,26,27].

In this context, patients’ eHealth literacy levels may influence their efficiency in accessing care resources, obtaining medical support, and engaging in chronic disease management. Diabetes patients are predominantly middle-aged and elderly individuals, who often have insufficient eHealth literacy [28]. This frequently leads to difficulties in online communication and underutilization of digital resources, which not only reduces the quality of chronic care but also hinders patients’ ability to translate their health needs into proactive self-management behaviors [29]. Prior studies have established a direct relationship between eHealth literacy and quality of chronic care [30], between eHealth literacy and self-management [31], and between quality of chronic care and self-management [32]. However, these studies have largely examined these relationships in isolation, without integrating the three into a single framework for comprehensive testing.

According to social cognitive theory, personal factors not only directly influence behavioral choices but also affect how individuals perceive and utilize environmental resources. Environmental feedback, in turn, may positively or negatively regulate individual behaviors [33]. This theoretical framework provides a rationale for conceptualizing the quality of chronic care as a mediator between eHealth literacy and self-management. Within this framework, eHealth literacy is a personal factor that reflects an individual’s ability to access and use digital health information in their information environment [34]. The quality of chronic care is considered an environmental resource, reflecting the medical resources and support that patients subjectively perceive within their care model [21]. Together, these concepts may illustrate how personal capabilities and environmental support jointly influence patients’ self-management behaviors. Thus, this study aimed to examine whether the quality of chronic care serves as a statistically significant mediator in the relationship between eHealth literacy and self-management among adults with diabetes in a shared care model. Specifically, we put forth the following hypotheses:

**H1:**  *eHealth literacy is positively related to diabetes self-management.*

**H2:**  *Quality of chronic care is positively related to diabetes self-management.*

**H3:**  *Quality of chronic care statistically mediates the relationship between eHealth literacy and diabetes self-management.*

## 2. Materials and Methods

### 2.1. Study Participants and Setting

This cross-sectional study enrolled patients with diabetes managed under the shared care model, selected via convenience sampling from a tertiary hospital in Northwest China from March 2025 to March 2026. Eligible participants were those aged ≥ 18 years with a confirmed diabetes diagnosis according to the criteria of the Chinese Diabetes Society guidelines [11], who were under the shared care model for diabetes management and provided written informed consent. Those who had gestational diabetes mellitus, psychiatric disorders or cognitive impairments that seriously affect communication, or hearing or speech disorders were excluded.

The shared care model is a comprehensive, whole-course diabetes management system that integrates online and offline approaches with multidisciplinary collaboration. Grounded in Diabetes Self-Management Education and Support (DSMES), this model bridges in-hospital treatment with out-of-hospital management, forming a continuous closed-loop care system. The multidisciplinary team consists of physicians, nurses, dietitians, and health managers, who work collaboratively to develop personalized medication, dietary, and exercise plans based on clinical guidelines and remind patients to undergo regular complication screenings. In terms of the management process, patients return to the hospital for follow-up visits and laboratory tests every quarter. Outside the hospital, they maintain online communication with the team via the “Shared Care” app, uploading self-management data such as blood glucose and dietary records. The care team then performs remote monitoring and provides tailored guidance based on these data. Unlike the traditional single-visit, passive care model, the shared care model leverages an information platform to extend management beyond the hospital setting, achieving active and continuous whole-course management through regular follow-up and sustained self-management support.

**Figure 1:**
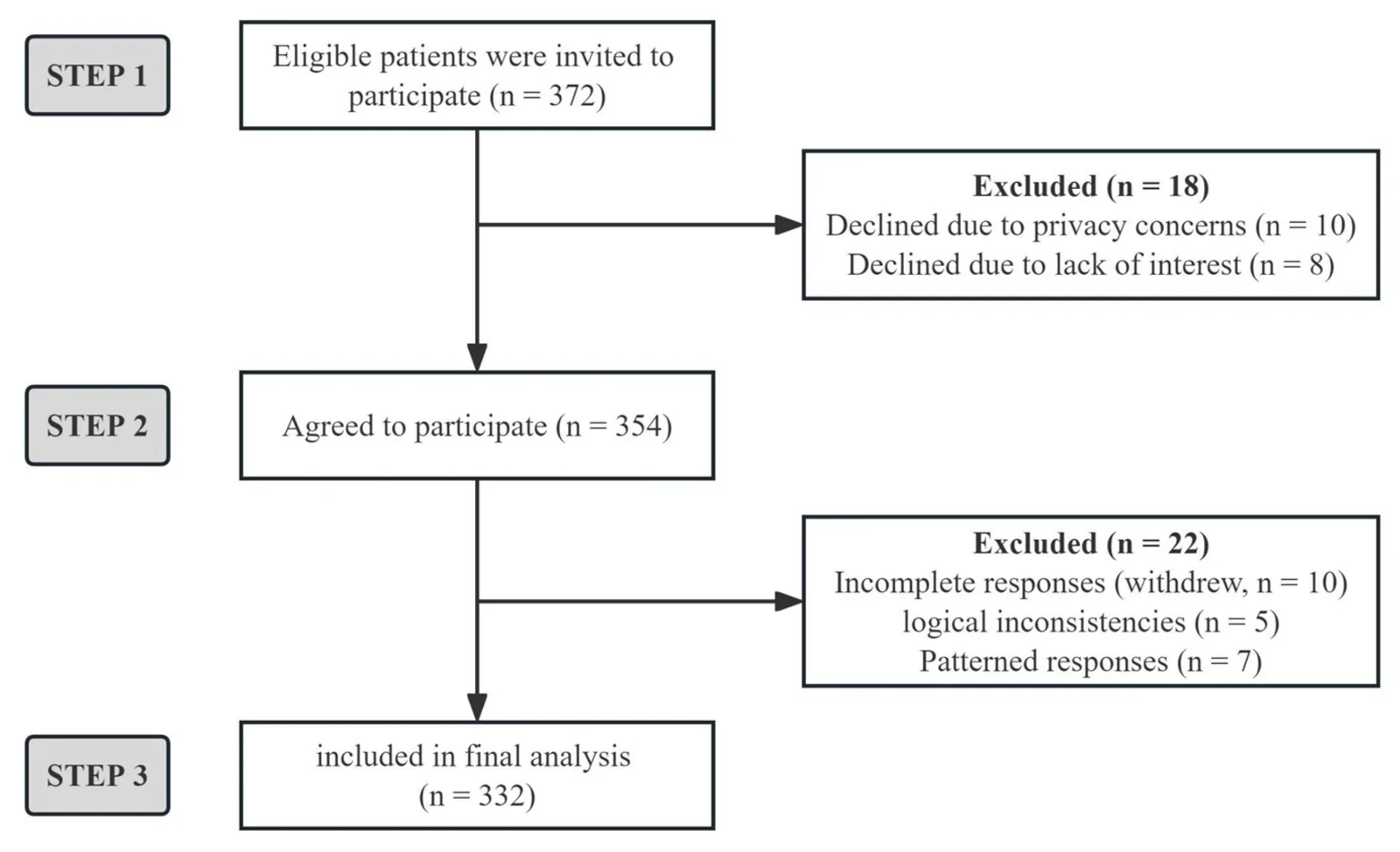
Participant flow diagram.

A priori power analysis using G*Power 3.1 with the default setting for a medium effect size (f^2^ = 0.15), α = 0.05, and power = 0.95 indicated a required minimum sample of 194 participants. A total of 372 patients with diabetes who met the inclusion criteria were approached, of whom 354 agreed to participate in the study. During recruitment, 10 participants withdrew for personal reasons. Subsequently, 5 questionnaires with logical inconsistencies (e.g., contradictory responses to reverse-coded items on the same scale) and 7 with patterned responses (e.g., identical choices throughout or fixed alternating patterns) were excluded, leaving 332 valid questionnaires included in the final analysis. The valid response rate was 93.79%. Figure 1 presents the participant screening and selection process.

### 2.2. Measures

#### 2.2.1. Participants’ Characteristics

General characteristics were obtained through a customized basic information questionnaire to gather participants’ baseline demographic and disease-related data. Baseline information included gender, age, body mass index (BMI), smoking habits, drinking status, and waist-to-hip ratio (WHR). Disease-related data included duration of diabetes, diabetic complications, family history, presence of hypertension, presence of hyperlipidemia, and duration of participation in the shared care model.

#### 2.2.2. eHealth Literacy Measurement

This study employed the eHealth Literacy Scale (eHEALS) to measure eHealth literacy. This scale was created by Norman [13], and the Chinese adaptation was provided by Guo et al. [35]. There are 8 items in the scale, each of which is measured on a 5-point Likert scale. The aggregate score spans from 8 to 40, with higher scores indicating greater eHealth literacy. Cronbach’s α was 0.974 in this study.

#### 2.2.3. Quality of Chronic Care Measurement

To evaluate quality of chronic care, the Patient Assessment of Chronic Illness Care (PACIC), initially designed by Glasgow et al. [21], was employed. The Chinese version of PACIC has 20 items, each measured on a 5-point Likert scale. The aggregate score spans from 20 to 100, with higher scores suggesting better quality of chronic care. As a self-report tool, the PACIC evaluates patients’ subjective perceptions of care provided in alignment with the CCM, but these perceptions may diverge from the actual care delivered under the care model. Cronbach’s α was 0.963 in this study.

#### 2.2.4. Self-Management Measurement

The Summary of Diabetes Self-Care Activities (SDSCA), designed by Toobert et al. [36] and adapted to the Chinese cultural context by Wan et al. [37], was employed to evaluate self-management. The SDSCA has 11 items measured on an 8-point Likert scale from 0 to 7. The aggregate score falls between 0 and 77, with higher scores reflecting better self-management behaviors. In this study, the Cronbach’s α was 0.820.

As the original versions of the three scales do not provide established cut-off points for classifying levels, the overall levels of the three variables in this study were interpreted relative to the theoretical midpoint of each scale. Scores above the midpoint were considered relatively high, while scores below the mid-point were considered relatively low. This approach has been used in previous studies when validated cut-off values were not available [38,39].

### 2.3. Procedure

Recruitment and data collection were conducted face-to-face in the outpatient clinic by four trained researchers using paper-based questionnaires. Before questionnaire administration, researchers assessed patients’ eligibility and whether they had previously participated in this study through face-to-face communication combined with electronic medical records. For eligible patients, researchers provided detailed information about the study purpose, procedures, and confidentiality measures and obtained written informed consent. After confirming a patient’s formal participation, researchers administered the paper-based questionnaire and extracted general demographic and clinical data from the electronic medical record system. No time limits were imposed for questionnaire completion, and researchers did not intervene in participants’ responses to the items. Completed questionnaires were returned to the researcher on-site. Participants were allowed to leave only after on-site verification confirmed no missing responses. Where omissions occurred, participants were requested to complete the questionnaire on the spot. During data cleaning, any duplicate entries were excluded. The study was approved by the Ethics Committee of the First Affiliated Hospital of Xi’an Jiaotong University (Approval No.: KYLLSL-2024-675-03) and followed the principles of the Declaration of Helsinki.

### 2.4. Statistical Analyses

All statistical procedures were implemented via SPSS 27.0. Descriptive analyses were conducted to summarize demographic data. Before analysis, the dataset was screened for missing values, and cases with incomplete responses were excluded to ensure data quality. The approximate normality of the data was examined using skewness and kurtosis, with values within ±3 for skewness and ±8 for kurtosis considered acceptable for parametric analysis. Group comparisons were performed using the independent-samples *t*-test (two groups) or one-way ANOVA (≥3 groups). For non-normally distributed data, the Kruskal–Wallis test was applied for multi-group comparisons. Pearson’s or Spearman’s correlation was used to assess associations among the core variables. Mediation was tested using Hayes’ PROCESS v4.1 (Model 4) with 5000 bootstrap resamples for bias-corrected 95% confidence intervals of indirect effects. The statistical significance threshold was defined as α = 0.05.

## 3. Results

### 3.1. Demographic Characteristics

A total of 332 participants were included in the final analysis, with an average age of 52.86 ± 14.05 years, and they were predominantly middle-aged or older adults. Table 1 presents the analysis results, indicating that participants with a family history of diabetes and those with shorter duration of participation exhibited greater diabetes self-management capabilities (both *p* < 0.05).

### 3.2. Descriptive Statistics of Core Variables

The descriptive statistics for the three core variables are presented in Table 2. The mean score of quality of chronic care was 89.82 ± 18.16, which was substantially above the theoretical midpoint of 60, indicating a relatively high level of perceived quality of chronic care among participants. A notable ceiling effect was observed, with 42.17% of the participants achieving the maximum possible score. The mean eHealth literacy score was 25.12 ± 8.20, slightly above the theoretical midpoint of 24, suggesting a moderate level of eHealth literacy. The mean self-management score was 48.73 ± 13.90, which was above the theoretical midpoint of 38.5, reflecting a relatively favorable level of self-management behaviors. All measured variables had absolute skewness values under 3 and absolute kurtosis values under 8, satisfying parametric assumptions for Pearson’s correlation analysis [40].

### 3.3. Variable Correlation Analysis

Pearson’s correlation analysis revealed that eHealth literacy was positively correlated with the quality of chronic care (r = 0.391, *p* < 0.01) and self-management (r = 0.322, *p* < 0.01), and the quality of chronic care was positively correlated with self-management (r = 0.374, *p* < 0.01). The absolute values of all correlation coefficients remained under the common cutoff of 0.7, which rules out severe multicollinearity among the variables. To further confirm this, the variance inflation factors (VIFs) were calculated, with all values staying below 5, supporting the above conclusion [41] (Table 3).

### 3.4. Statistical Mediating Role of Quality of Chronic Care

**Figure 2:**
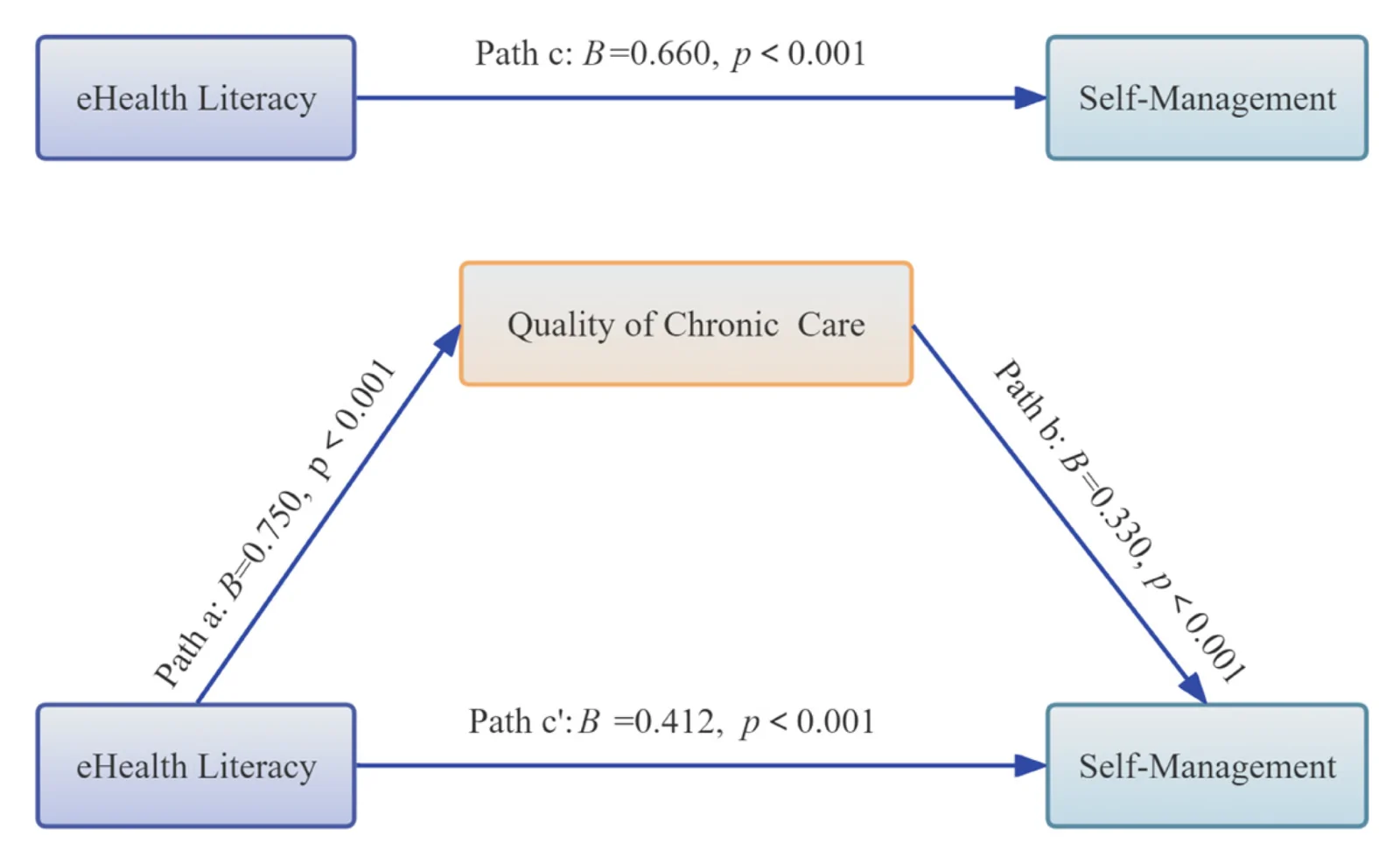
Statistical mediation model examining the indirect association between eHealth literacy and self-management through quality of chronic care.

This study employed the PROCESS macro Model 4 for the mediation analysis. As shown in Table 4, variables with statistical significance in the univariate analysis were included as control variables in the mediation model. The results indicated that eHealth literacy was positively associated with self-management (*B* = 0.660, *p* < 0.001). After entering the quality of chronic care as a statistical mediator into the model, eHealth literacy maintained a significant direct association with self-management (*B* = 0.412, *p* < 0.001). Therefore, hypotheses H1 and H2 were supported. The corresponding mediation model is presented in Figure 2, with eHealth literacy serving as the independent variable, quality of chronic care as the statistical mediator, and self-management as the dependent variable.

### 3.5. Pathway Modeling and Mediation Analysis

As shown in Table 5, using the bootstrap procedure to examine the statistical mediating role, we found that the indirect association of the quality of chronic care was β = 0.146 (95% CI: 0.114–0.398). The confidence interval excluded zero. This suggests that the quality of chronic care statistically mediated the relationship between eHealth literacy and self-management, and the statistical indirect effect contributed 35.53% to the overall effect. Therefore, H3 was supported. It should be noted that this proportion represents only a descriptive decomposition of associations based on cross-sectional data and does not support causal inference.

## 4. Discussion

Diabetes is a lifelong metabolic disease for which self-management is central to disease control. Therefore, elucidating the determinants and potential pathways of self-management behaviors is clinically valuable for developing effective interventions. We found significant positive correlations among patients’ levels of self-management, the quality of chronic care, and eHealth literacy. Furthermore, the quality of chronic care statistically mediated the relationship between eHealth literacy and self-management.

The findings suggest that patients with diabetes participating in the shared care model reported self-management behavior scores higher than those published in the previous literature [42]. This difference may be related to differences in the age and geographic region among the diabetes patients, as well as to the patients’ participation in the shared care model. Furthermore, patients with a family history of diabetes reported higher levels of self-management, in line with earlier research [43]. Such patients may have had long-term exposure to disease-related information and self-management experience [44]. This exposure may contribute to a clearer understanding of the harm associated with diabetes and the acquisition of specific management experiences and behavioral models from affected relatives, which may facilitate more proactive self-management behaviors [45]. An unexpected finding was that patients who had participated in the shared care model for more than six months had significantly lower levels of self-management than those who had participated for six months or less. This result is consistent with a six-month longitudinal follow-up study, which observed a gradual decline in patients’ self-management behaviors [46]. This difference may be related to additional burdens faced by long-term participants, including disease management fatigue and increased complications, which may contribute to a decline in self-management capacity [47]. Moreover, patients with poorer glycemic control or lower self-management capacity may be more likely to remain in the model [48,49], which may partly account for the observed difference. In this study, overweight and obese patients with diabetes accounted for 35.54% and 25.90%, respectively, of the cohort. Although the difference in self-management scores between these groups did not reach statistical significance, being overweight or obese is known to increase the treatment burden and management complexity [50], which may pose challenges to diabetes self-management [51,52]. These findings suggest that weight management should be considered a potential component of diabetes self-management support, particularly given the high prevalence of overweight and obesity in this population.

As we hypothesized, eHealth literacy was positively associated with self-management, a finding that is in line with prior research [31]. This association may reflect that the widespread availability of digital resources has profoundly transformed health communication patterns and patients’ health-related behaviors [53]. Specifically, higher eHealth literacy may enable individuals to access and apply digital health information and services more effectively, which may contribute to greater health benefits [54]. Nevertheless, although patients prefer to seek health information through digital technologies, their insufficient eHealth literacy may result in persistently low utilization rates [55]. This is primarily due to persistent inequalities in the access to and use of digital resources, a disparity often referred to as the digital divide [56]. Low eHealth literacy may reduce patients’ motivation to utilize digital health resources, potentially contributing to differences in self-management behaviors and impeding standardized disease management [57]. This finding suggests that systematic digital skills training programs may be a useful strategy to help patients with diabetes both improve their eHealth literacy and better navigate digital health resources [58], which may in turn improve their self-management capabilities.

Although the association between eHealth literacy and self-management has previously been examined, few studies have explored the mediating role of quality of chronic care in this relationship among patients managed under a shared care framework. In this study, we found that eHealth literacy was indirectly associated with self-management through quality of chronic care. The indirect association accounted for 35.53% of the total association, indicating a partial and statistically significant mediating role. This finding may reflect the fact that healthcare services delivered through digital health technologies could play a role in the transformation of health knowledge into health-promoting behaviors. With the widespread use of digital healthcare, patients with higher eHealth literacy are more inclined to actively acquire and integrate disease-related knowledge [58]. At the same time, they are better able to perceive and utilize the services and resources provided by the chronic care model and apply these resources to the daily management of their disease [59]. Furthermore, the internet-based chronic care model may have reshaped the doctor–patient communication patterns, allowing patients to express their needs regardless of time or location, obtain timely feedback, and participate more actively in health-related decisions [60]. Such positive care experiences may enhance patients’ recognition of the shared care model and ultimately contribute to standardized and beneficial self-management behaviors. In addition, the duration of participation was negatively associated with self-management but positively associated with quality of chronic care. The opposite directions of these two pathways may reflect that patients’ self-management capacity declines over time and, thus, they require longer care support [61]. Meanwhile, longer participation in care services may strengthen their perceived support, which may be associated with more favorable evaluations of care quality [62]. A family history of diabetes was not significantly associated with self-management in the final model, suggesting that compared with genetic background, modifiable care factors may be more relevant to improving self-management. However, the full mediation model explained only 41.7% of the variance in self-management, indicating that other unmeasured factors may also contribute to self-management behaviors. Previous studies have found that socioeconomic status and social support may play important roles in self-management [63,64], and future research could further examine these variables within the present framework. Meanwhile, the observed ceiling effect in the quality of chronic care scores may have limited their variability, potentially affecting the estimation of the indirect association. Therefore, the mediation findings should be interpreted with caution.

The above findings offer several potential implications for clinical practice. First, chronic care teams may consider integrating eHealth literacy assessment into routine diabetes care. For patients with low eHealth literacy, chronic care teams may provide tiered digital health training tailored to their age and cognitive characteristics to help them more fully integrate the online resources and remote support offered by the care model into their daily self-management [65,66]. Second, healthcare institutions may consider optimizing the patient-centered chronic care model, with particular attention to long-term participants. In practice, identifying patients’ needs at different participation stages and developing flexible, individualized care strategies may enhance their care experiences and perceptions of care quality, thereby better supporting self-management. Future intervention may help determine whether these strategies are effective in improving self-management among diabetes patients.

We acknowledge several limitations of the present study. First, the cross-sectional design limits causal inference, and reverse causality cannot be excluded. For instance, patients with better self-management ability may be more inclined to actively participate in the shared care model and make better use of available resources, which may be associated with more positive evaluations of the quality of chronic care. Therefore, future prospective longitudinal studies are recommended to determine the temporal sequence and causal directions among these variables. Second, data were collected from only one institution in China through convenience sampling, which constrains the generalizability of the results. Subsequent multicenter studies with random sampling designs will help improve the external validity of the results. Third, the quality of chronic care scores in this study tended to cluster at the higher end among participants, suggesting a possible ceiling effect that may limit the ability to distinguish variations in the quality of chronic care. In future research, the use of more refined measurement instruments or the incorporation of objective care quality indicators may be considered to more comprehensively capture the variability in chronic illness care quality. Finally, all variables in this study were collected through self-report measures, which may introduce common method variance and potentially inflate the associations among variables. This methodological limitation suggests that caution should be exercised when interpreting the findings of this study. Future research could employ designs such as multi-time-point measurements or multimodal data collection to reduce the potential impact of common method bias on research conclusions.

## 5. Conclusions

Among diabetic patients within the shared care model, self-management and the quality of chronic care were at relatively favorable levels, while eHealth literacy was at a moderate level. eHealth literacy was positively associated with self-management, and the quality of chronic care played a partial and statistically significant mediating role in this relationship. However, due to the cross-sectional design, the mediation findings should not be interpreted as causal. That is, a higher quality of chronic care does not necessarily predict better self-management.

## Figures and Tables

**Table 1:** Characteristics and self-management differences in participants (N = 332).

Variables	Mean ± SD/n (%)	Self-Management (Score)	*p*-Value
Gender			0.582 ^a^
Male	198 (59.64)	49.08 ± 13.59	
Female	134 (40.36)	48.22 ± 14.39	
Age (years)	52.86 ± 14.05		0.371 ^a^
<45	99 (29.80)	47.69 ± 13.19	
≥45	233 (70.20)	49.18 ± 14.20	
Disease duration (years)			0.680 ^a^
<10	215 (64.80)	48.50 ± 14.12	
≥10	117 (35.20)	49.16 ± 13.54	
BMI (kg/m^2^)			0.087 ^b^
<18.5	5 (1.51)	52.50 (47.25~64.50)	
18.5~	123 (37.05)	50.00 (43.00~62.00)	
24.0~	118 (35.54)	47.50 (37.25~56.00)	
≥28.0	86 (25.90)	47.00 (35.00~56.00)	
WHR	0.952 ± 0.078	48.730 ± 13.903	0.345 ^c^
Duration of participation (months)			<0.001 ^a^
≤6	131 (39.50)	54.44 ± 17.72	
>6	201 (60.50)	45.02 ± 8.98	
Drinking status			0.383 ^a^
No	278 (83.73)	49.03 ± 13.75	
Yes	54 (16.27)	47.22 ± 14.72	
Smoking habits			0.433 ^a^
No	270 (81.33)	49.02 ± 13.90	
Yes	62 (18.67)	47.48 ± 13.98	
Diabetic complications			0.339 ^a^
No	291 (87.65)	48.46 ± 14.04	
Yes	41 (12.35)	50.68 ± 12.85	
Family history of diabetes			0.003 ^a^
No	203 (61.14)	46.91 ± 14.59	
Yes	129 (38.86)	51.61 ± 12.36	
Hypertension comorbidity			0.061 ^a^
No	268 (80.72)	49.43 ± 13.61	
Yes	64 (19.28)	45.81 ± 14.80	
Hyperlipidemia comorbidity			0.766 ^a^
No	290 (87.35)	48.65 ± 14.01	
Yes	42 (12.65)	49.33 ± 13.29	

Note: ^a^ analyzed using independent samples *t*-test; ^b^ analyzed using the Kruskal–Wallis *H* test; ^c^ analyzed using Pearson’s correlation.

**Table 2:** Descriptive statistics of core variables (N = 332).

Variables	M ± SD	Min	Max	Scale MidPoint	Skewness	Kurtosis	Ceiling (%)
Quality of chronic care	89.82 ± 18.16	20	100	60.0	−2.78	7.65	42.17
eHEALS	25.12 ± 8.20	8	40	20.0	−0.91	−0.07	1.20
SDSCA	48.73 ± 13.90	14	77	38.5	−0.40	0.02	0.60

**Table 3:** Correlation analysis for core study variables (N = 332).

Variables	eHEALS	Quality of Chronic Care	SDSCA	VIF
eHEALS	-			1.180
Quality of chronic care	0.391 **	-		1.180
SDSCA	0.322 **	0.374 **	-	-

Note: ** *p* < 0.01.

**Table 4:** Assessment of the mediating role of quality of chronic care (M) on eHealth literacy (X) and self-management (Y) (N = 332).

Variables		X-Y			X-M			X-M-Y		
		B	SE	*t*-Value	B	SE	*t*-Value	B	SE	*t*-Value
Independent variable	X	0.660	0.081	8.119 ***	0.750	0.109	6.857 ***	0.412	0.078	5.288 ***
Mediator variable	M	–	–	–	–	–	–	0.330	0.037	8.960 ***
Control variables	C_1_	−11.093	1.368	−8.111 ***	9.856	1.840	5.357 ***	−14.346	1.280	−11.208 ***
	C_2_	3.178	1.350	2.355 *	4.670	1.816	2.572 *	1.637	1.223	1.339
Model fit	*R*	0.523			0.479			0.645		
	*R* ^2^	0.273			0.230			0.417		
	*F*	41.128 ***			32.578 ***			58.372 ***		

Note: Unstandardized *B* coefficients are provided; “–“ means omission of the variable from that step; X: eHealth literacy; M: quality of chronic care; Y: self-management; C_1_: duration of participation; C_2_: family history of diabetes; * *p* < 0.05; *** *p* < 0.001.

**Table 5:** Testing the statistical significance of the mediating role (n = 332).

Path	B	SE	β	*p*-Value	95%CI	Coefficients
Total pathway	0.660	0.081	0.389	<0.001	0.500–0.819	-
Direct pathway	0.412	0.078	0.243	<0.001	0.259–0.566	62.42%
Indirect pathway	0.248	0.073	0.146	<0.050	0.114–0.398	37.58%

## Data Availability

Due to concerns regarding participants’ personal privacy and restrictions imposed by ethical approval, the data used in this study are not publicly available. However, the datasets analyzed during the current study can be accessed by contacting the corresponding author upon reasonable request.

## Abbreviations

The following abbreviations are used in this manuscript:

BMI	Body Mass Index
CCM	Chronic Care Model
eHEALS	eHealth Literacy
WHR	Waist-to-Hip Ratio
SDSCA	Summary of Diabetes Self-Care Activities
